# Blockade of innate inflammatory cytokines TNF**α**, IL-1**β**, or IL-6 overcomes virotherapy-induced cancer equilibrium to promote tumor regression

**DOI:** 10.1093/immadv/ltad011

**Published:** 2023-07-03

**Authors:** Michael J Walsh, Lestat R Ali, Patrick Lenehan, Courtney T Kureshi, Rakeeb Kureshi, Michael Dougan, David M Knipe, Stephanie K Dougan

**Affiliations:** Department of Microbiology, Blavatnik Institute, Harvard Medical School, Boston, MA, USA; Harvard Program in Virology, Boston, MA, USA; Department of Cancer Immunology and Virology, Dana-Farber Cancer Institute, Boston, MA, USA; Division of Gastroenterology, Department of Medicine, Massachusetts General Hospital, Boston, MA, USA; Department of Cancer Immunology and Virology, Dana-Farber Cancer Institute, Boston, MA, USA; Department of Medicine, Harvard Medical School, Boston, MA, USA; Department of Cancer Immunology and Virology, Dana-Farber Cancer Institute, Boston, MA, USA; Department of Immunology, Harvard Medical School, Boston, MA, USA; Department of Cancer Immunology and Virology, Dana-Farber Cancer Institute, Boston, MA, USA; Division of Gastroenterology, Department of Medicine, Massachusetts General Hospital, Boston, MA, USA; Department of Immunology, Harvard Medical School, Boston, MA, USA; Department of Cancer Immunology and Virology, Dana-Farber Cancer Institute, Boston, MA, USA; Department of Immunology, Harvard Medical School, Boston, MA, USA; Division of Gastroenterology, Department of Medicine, Massachusetts General Hospital, Boston, MA, USA; Department of Medicine, Harvard Medical School, Boston, MA, USA; Department of Microbiology, Blavatnik Institute, Harvard Medical School, Boston, MA, USA; Department of Cancer Immunology and Virology, Dana-Farber Cancer Institute, Boston, MA, USA; Department of Immunology, Harvard Medical School, Boston, MA, USA

**Keywords:** equilibrium, cytokine blockade, macrophage, TNF, IL6, IL1B

## Abstract

Cancer therapeutics can lead to immune equilibrium in which the immune response controls tumor cell expansion without fully eliminating the cancer. The factors involved in this equilibrium remain incompletely understood, especially those that would antagonize the anti-tumor immune response and lead to tumor outgrowth. We previously demonstrated that continuous treatment with a non-replicating herpes simplex virus 1 expressing interleukin (IL)-12 induces a state of cancer immune equilibrium highly dependent on interferon-γ. We profiled the IL-12 virotherapy-induced immune equilibrium in murine melanoma, identifying blockade of innate inflammatory cytokines, tumor necrosis factor alpha (TNFα), IL-1β, or IL-6 as possible synergistic interventions. Antibody depletions of each of these cytokines enhanced survival in mice treated with IL-12 virotherapy and helped to overcome equilibrium in some tumors. Single-cell RNA-sequencing demonstrated that blockade of inflammatory cytokines resulted in downregulation of overlapping inflammatory pathways in macrophages, shifting immune equilibrium towards tumor clearance, and raising the possibility that TNFα blockade could synergize with existing cancer immunotherapies.

## Graphical Abstract

**Graphical Abstract:**
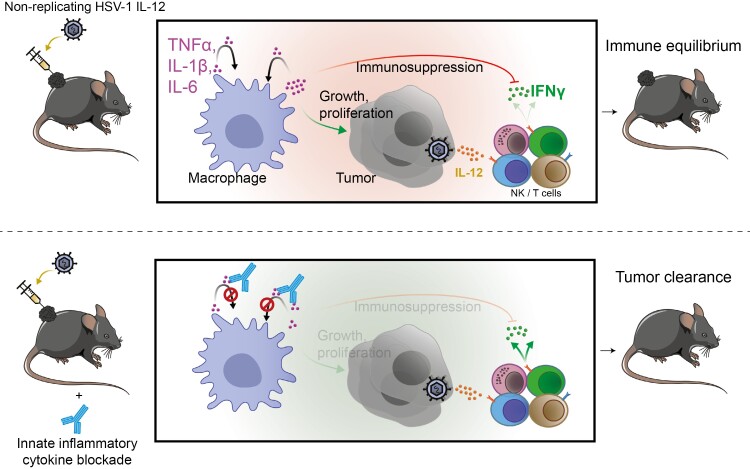

## Introduction

In humans and mice, immunity plays a role in the elimination, control, and even outgrowth of tumors in a process known as cancer immunoediting [1, 2]. Equilibrium, or the balance between tumor immune control and tumor outgrowth, often occurs in clinically undetected tumors, both on and off therapy, which makes studying this state in patients very difficult. Despite the success of checkpoint inhibitors inducing durable responses through the targeting of cytotoxic T-lymphocyte-associated protein 4 (CTLA-4) and programmed cell death protein/ligand 1 (PD-1/L1), some patients with stable or otherwise undetectable disease can relapse and suddenly lose tumor control [3, 4]. Immunosuppressive myeloid cells may counteract tumor-specific T cells, partly explaining failure of the immune system to fully clear equilibrium-state tumors and eventually accounting for tumor outgrowth [5]. However, it is not fully clear what factors might tip the balance in favor of tumor rejection or outgrowth.

We previously demonstrated that local delivery of interleukin-12 (IL-12) with a replication-defective herpes simplex virus 1 (HSV-1) vector, *d*106S-IL12, led to prolonged survival of mice with melanoma and induction of an immune equilibrium state with long-term treatment [6]. Here, we used our model of therapy-induced immune equilibrium to test what pro-tumor factors may prevent full cancer clearance. We analyzed the cytokine milieu of the tumor microenvironment and discovered high levels of the pro-inflammatory cytokines tumor necrosis factor (TNF)-α, interleukin (IL)-1β, and IL-6. The role that these cytokines, especially TNFα, play in cancer immune responses is still controversial [7]. All three innate inflammatory cytokines are important for promoting oncogenesis, but their role in established tumors is less clear [8–10]. Here we demonstrate that they aid in maintaining tumors in equilibrium, and may eventually help tumors to escape from immune control. Blocking each of these cytokines helped overcome the equilibrium state in some mice and led to complete eradication of their tumors. Through single-cell RNA-sequencing, we identified overlapping inflammatory and immunosuppressive pathways that were downregulated with blockade of each pro-inflammatory cytokine. As each of these cytokine-blocking therapies is already approved for clinical use, these results suggest the potential of pro-inflammatory cytokine blockade in treating patients with cancer undergoing immunotherapy.

## Results

### Intratumoral d106S-IL12 injection results in a large increase in pro-inflammatory cytokines

**Figure 1.**
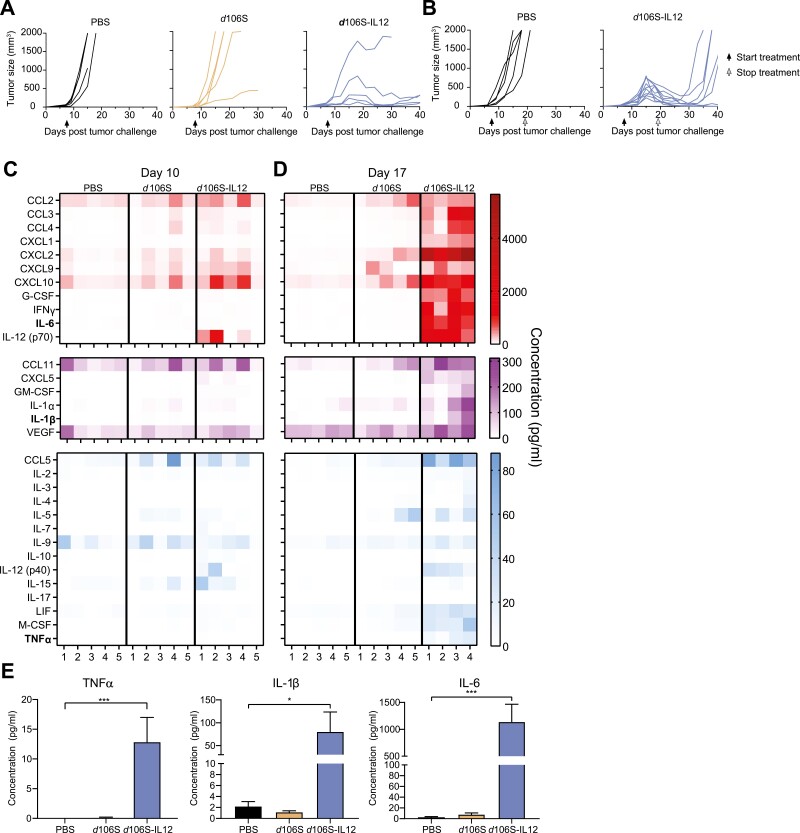
Injection of *d*106S-IL12 virus generates immune equilibrium and results in pro-inflammatory cytokine secretion. (A) Mice bearing B16^Nectin1^ tumors were injected intratumorally every 3 days starting on day 7 with the listed treatments (*N* = 5 mice per group). (B) Mice were treated as in (A), but therapy was stopped at day 19 after five total injections (*N* = 5/10 mice for PBS/*d*106S-IL12). (C/D) Following seven days of B16^Nectin1^ tumor growth, mice were injected intratumorally every three days with the listed treatments. For better visualization, cytokines are grouped based on high, medium, and low concentrations present in the tumor. (C) At day 10 post-tumor challenge, following one injection or (D) at day 17 post-tumor challenge, following four injections, mice were sacrificed. Tumor lysates were subjected to cytokine/chemokine analysis by multiplex array. Protein lysates were normalized using a BCA assay. Each column represents one mouse (*N* = 5 mice per group, except *N* = 4 for *d*106S-IL12 day 17). (E) Data from (D) day 17 cytokine levels. Groups were compared with a one-way ANOVA and Dunnett’s multiple comparisons test; *P < 0.05, ***P < 0.001.

We previously established the use of continuous periodic injections of *d*106S-IL12 as a mouse model of cancer immune equilibrium [6]. We generated B16^Nectin1^ cells, which, unlike wild-type B16, are permissive to HSV-1 entry [6], and injected mice bearing these tumors intratumorally with various treatments. As seen previously [6], continuous treatment with *d*106S-IL12 starting at day 7 resulted in the majority of mice entering an equilibrium state where the tumors neither grew out nor were fully rejected (Fig. 1A). Injection of the empty viral vector alone was insufficient to generate immune equilibrium (Fig. 1A). Continuous therapy was also necessary to induce equilibrium; virotherapy cessation at day 19 resulted in escape from tumor control (Fig. 1B), as observed previously [6].

To examine the microenvironment of these tumors, we monitored the presence of cytokines induced by virotherapy *in vivo*. After one week of tumor growth, mice were injected once intratumorally with PBS, *d*106S, or *d*106S-IL12. At 3 days following a single intratumoral injection (10 days post-tumor challenge) or after four intratumoral injections (17 days post-tumor challenge), we examined cytokine/chemokine protein levels in tumor lysates. While there were some cytokine changes at day 10, by day 17 d106S-IL12 injections resulted in an elevation of many cytokines compared to PBS or *d*106S treatment (Fig. 1C and D). Downstream targets of IL-12, such as interferon (IFN)γ [11], were highly elevated by day 17 (Fig. 1C and D). These findings aligned with our previous findings on the centrality of IFNγ in establishing and maintaining equilibrium [6].

We also noted a large increase in myeloid-recruiting cytokines and chemokines that might be driving an immunosuppressive, pro-tumor microenvironment. Chemokines and growth factors that increased included CCL2, CXCL1, CXCL2, and G-CSF, some of which were secreted up to almost 100-fold more than on day 10 (Fig. 1C and D). Of note, the pro-inflammatory cytokines TNFα, IL-1β, and IL-6 also increased between day 10 and day 17 (Fig. 1E). We hypothesized that innate inflammatory cytokines would help initiate recruitment of suppressive myeloid cells and enhance their suppressive functions. All three cytokines are targets of monoclonal antibodies that are approved for use in various autoimmune diseases [8, 12, 13], and as such could potentially be combined with existing therapies for the treatment of cancer.

### Pro-inflammatory cytokine blockade helps to overcome equilibrium

To test the role of innate inflammatory cytokines in the establishment of immune equilibrium, we treated B16^Nectin1^ tumors with PBS or *d*106S-IL12 every 3 days starting at day 7 for a total of five injections. On day 10 post tumor challenge, when levels of the three pro-inflammatory cytokines were still low (Fig. 1C), we began injections of validated blocking antibodies targeting TNFα, IL-1β, IL-6R, or no blockade [14–16]. Blocking antibodies were injected every 3 days for a total of six injections. Our previous results (Fig. 1A and B) [6] demonstrated that escape from equilibrium would occur when therapy was halted. Therefore, any additional benefit or detriment from blocking these cytokines would be apparent with this short course of treatment.

**Figure 2.**
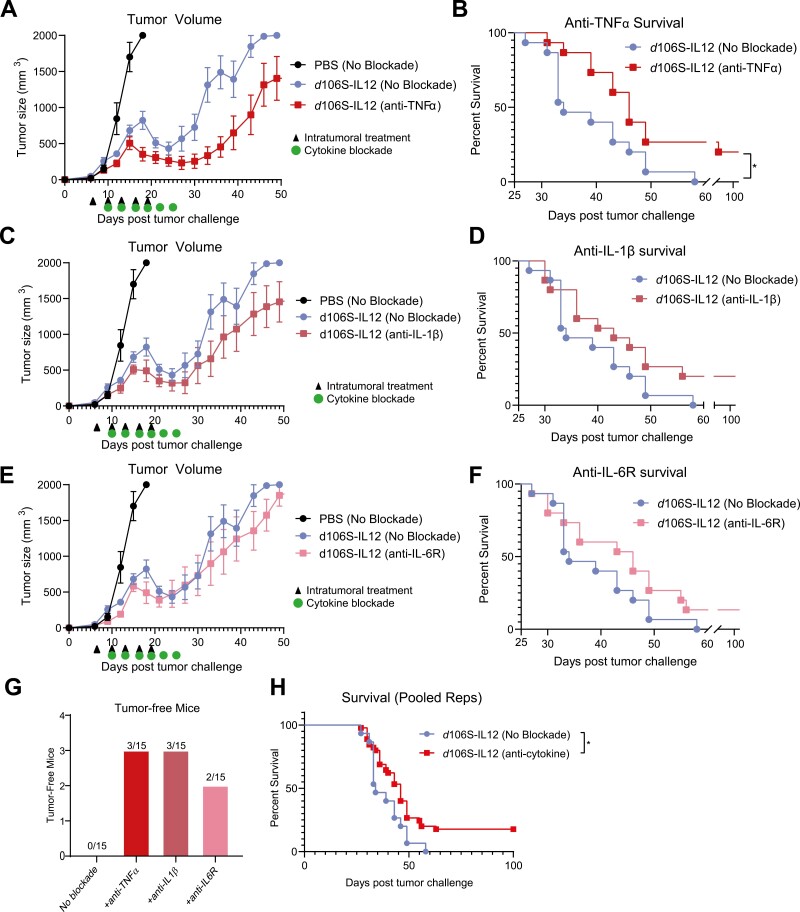
Pro-inflammatory cytokine blockade slows tumor growth and overcomes *d*106S-IL12-induced immune equilibrium. B16^Nectin1^ tumors were treated intratumorally starting at day 7 every 3 days for total of five injections (*N* = 5 for PBS, *n* = 10 for *d*106S-IL12 groups). At day 10, blocking antibodies against (A/B) TNFα, (C/D) IL-1β, or (E/F) IL-6R (100 µg per antibody) were injected intraperitoneally every 3 days for a total of six injections. Survival curves (B/D/F) are across two independent experiments (*N* = 15 mice per group). (G) Tumor-free mice from survival experiments of single cytokine blockade. (H) Pooled survival across two independent experiments (*N* = 15/45 for no blockade/cytokine blockade groups). Log-rank test; *P < 0.01. Arrows indicate intratumoral treatments (PBS/d106S-IL12), green circles indicate cytokine blockade.

Tumor volumes were reduced when *d*106S-IL12 treatment was combined with TNFα blockade (Fig. 2A), leading to significantly enhanced survival in mice (Fig. 2B). Mice treated with combination virotherapy and IL-1β or IL-6R blockade had slightly, but not significantly reduced tumor volumes and enhanced survival (Fig. 2C–F), which could potentially be attributed to the greater concentrations of IL-1β and IL-6 within the tumor being more difficult to fully block with therapeutic antibodies (Fig. 1E). Mice treated with just five injections of *d*106S-IL12 all eventually succumbed to their tumors. However, any individual inflammatory cytokine blockade combined with *d*106S-IL12 virotherapy resulted in several long-term surviving mice (8 out of 45) which were tumor-free without additional treatment (Fig. 2G). The combined survival of blocking any individual cytokine was significantly increased over no blockade (Fig. 2H). These results together demonstrated that innate inflammatory cytokines TNFα, IL-1β, and IL-6 contribute to immune suppression following IL-12 virotherapy and that their blockade could enhance the effect of an immunotherapy, helping to overcome equilibrium.

### Single-cell RNA sequencing of tumors undergoing IL-12 virotherapy and pro-inflammatory cytokine blockade reveals distinct changes in the microenvironment

**Figure 3.**
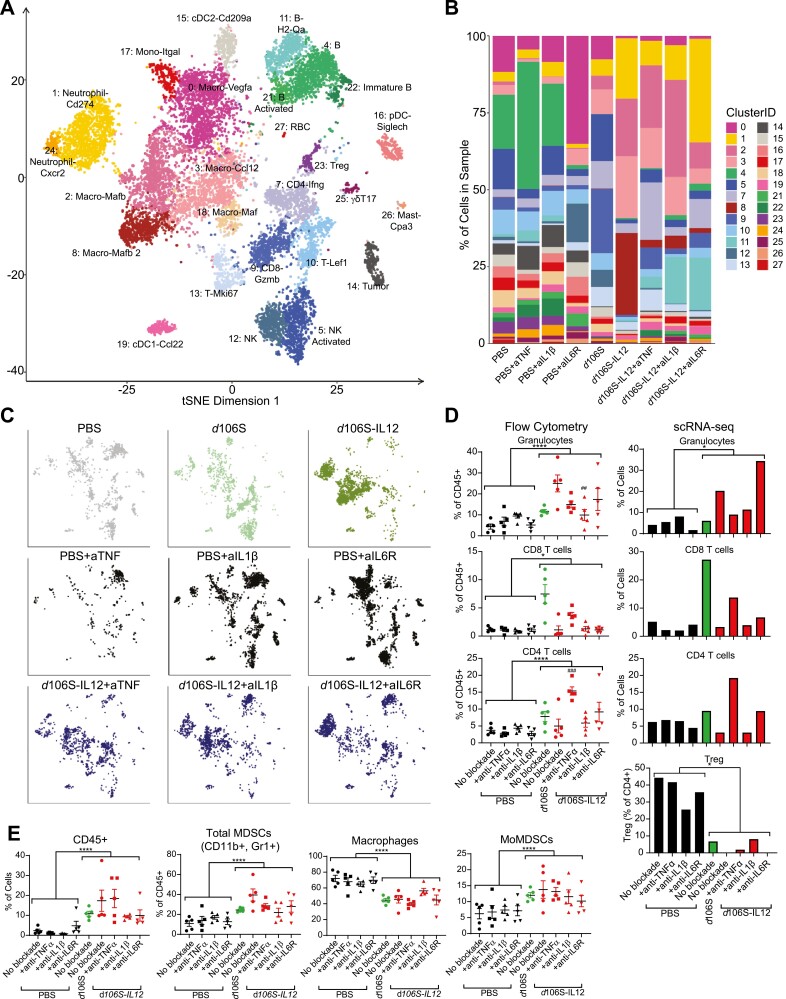
Cytokine blockade does not profoundly change levels of immune cells infiltrating tumors. B16^Nectin1^ tumors were treated intratumorally starting at day 7 every 3 days for total of four injections of either PBS, d106S, or d106S-IL12. At day 10, blocking antibodies against either TNFα, IL-1β, or IL-6R (100 µg per injection) were injected every 3 days for a total of three injections (*N* = 5 per treatment group). Mice were sacrificed on day 16 (PBS groups) or day 17 (*d*106S, *d*106S-IL12 groups) post-tumor challenge. Tumors from each treatment group were pooled (*N* = 5) and sorted for CD45+ cells and subjected to single-cell RNA-sequencing. (A) t-SNE plot of all analyzed cells colored by unsupervised clustering. (B) Cell cluster frequency within each treatment sample. (C) Distribution of cell clusters across treatment groups from (A). (D/E) Flow cytometry on individual tumor samples from the scRNA-seq. (D) Flow cytometry analysis was compared to scRNA-seq clustering showing similar patterns. Gating strategy in Supplementary Fig. 2. Wilcoxon signed rank test *P < 0.05 for scRNA-seq; unpaired t-test for pooled flow cytometry samples *P < 0.05, ***P < 0.001; one-way ANOVA and Tukey’s multiple comparisons test for *d*106S-IL12 no blockade vs. cytokine blockades ^##^P < 0.01, ^###^P < 0.001. This experiment was analyzed in part previously [6].

To understand the mechanisms by which innate inflammatory cytokine blockade overcomes immune equilibrium, we analyzed the tumors undergoing cytokine blockade by single-cell RNA-sequencing (scRNA-seq). Following a similar pattern of treatment as Fig. 2, we gave mice four intratumoral doses of PBS, *d*106S, or *d*106S-IL12 and three doses of either anti-TNFα, anti-IL-1β, anti-IL6R, or no blockade (*d*106S group did not receive blocking antibodies). At day 16 or 17 for PBS or virus-treated mice, respectively, we pooled tumors from each treatment type, isolated CD45^+^ cells, and analyzed them by single-cell RNA sequencing. Sequencing analysis of the groups lacking cytokine blockade was performed in our prior study [6]; here we also include samples that underwent cytokine blockade to understand its effects. Unsupervised clustering analysis revealed distinct groups of immune cells (Fig. 3A) classified based on expression of various genetic markers (Supplementary Fig. 1). When separated by cluster and treatment type, the frequency of cells present in each sample was heterogeneous but influenced primarily by the presence or absence of *d*106S-IL12 virotherapy. That is, virotherapy was inducing identifiable cell type changes compared to PBS treatments, but the changes based on cytokine blockade within *d*106S-IL12 groups were less obvious at the level of cell frequency (Fig. 3B and 3C). Individual tumors taken for single-cell analysis were also analyzed by flow cytometry. Flow cytometric analysis was also performed in our previous study [6] but we expand the findings here to include cytokine blockade groups. Comparisons of flow cytometric to scRNA-seq data showed similar trends in the clustering of cells by treatment type (Fig. 3D). Overall, by flow cytometry, most of the levels of infiltrating cells were not dramatically changed with cytokine blockade combined with *d*106S-IL12 therapy, compared to IL12 virotherapy alone. Compared to PBS groups, we saw an overall increase in granulocytes, as well as CD8^+^ and CD4^+^ T cells with any *d*106S vector treatment (Fig. 3D). The only significant changes occurring between cytokine blockade and no blockade in mice treated with *d*106S-IL12 were a decrease in granulocytes with IL-1β blockade and an increase in CD4^+^ T cells with TNFα blockade (Fig. 3D). However, we saw no increase in Tregs infiltrating tumors treated with TNFα blockade, suggesting that the increased CD4^+^ T cells are CD4 effector cells (Fig. 3D). All virus-treated samples had increased CD45^+^ cells present in their tumors (Fig. 3E). Tumors treated with virus also had an increase in myeloid-derived suppressor cells (MDSCs), including monocytic MDSCs (MoMDSCs) (Fig. 3E), consistent with increased myeloid-recruiting chemokines (Fig. 1E), and suggesting that these cell types infiltrating the tumor might be counteracting anti-tumor immunity. However, MDSC populations were not changed with cytokine blockade, and thus did not explain the improved survival.

### Blockade of any pro-inflammatory cytokine transcriptionally reprograms macrophages and regulates overlapping inflammatory pathways with other cytokines

**Figure 4.**
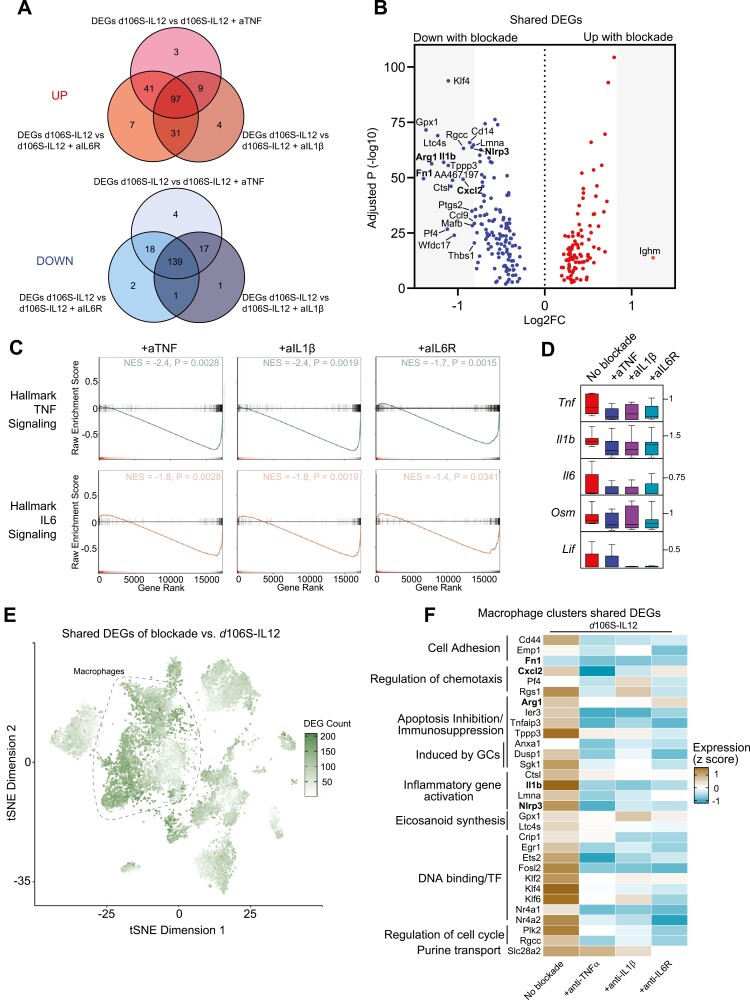
Pro-inflammatory cytokine blockades transcriptionally reprogram macrophages and reduce similar inflammatory pathway genes, regardless of targeted cytokine. (A) Differentially expressed gene (DEG) analysis was performed comparing *d*106S-IL12 no blockade to *d*106S-IL12 plus pro-inflammatory cytokine blockade groups (+anti-TNFα, IL-1β, or IL-6R) from the scRNA-seq data. Shown are Venn diagrams of shared DEGs among cytokine blockade groups. (B) Volcano plot of shared DEGs upregulated or downregulated by pro-inflammatory cytokine blockade. (C) Gene Set Enrichment Analysis for hallmark TNF or IL6 signaling and response genes for the three cytokine blockade groups (+*d*106S-IL12) versus d106S-IL12 no blockade. NES = normalized enrichment score. (D) Normalized expression of cytokines across top expressing clusters. (E) t-SNE plot where each cell is shaded according to the number of shared DEGs that it expresses. High expression of genes largely falls within the macrophage/monocyte clusters (see Fig. 3A). (F) Top significantly downregulated inflammatory genes in macrophage/monocyte clusters with greater than 0.6 average log-fold change versus no blockade *d*106S-IL12. Genes are grouped by function. GC = glucocorticoids, TF = transcription factor.

Because most cell populations did not change in frequency as a result of cytokine blockade, we hypothesized that transcriptional reprogramming would underlie the effect of cytokine blockade in enhancing *d*106S-IL12 responses. To this end, we performed differential expression analysis comparing the *d*106S-IL12 sample without cytokine blockade to each of the other cytokine blockades combined with *d*106S-IL12 treatment, which generated a list of significantly differentially expressed genes (DEGs) for each specific cytokine blocked. The majority of DEGs were shared between cytokine blockade treatments (Fig. 4A), suggesting that TNFα, IL-1β, and IL-6 signaling converge on similar pathways in the tumor microenvironment.

When we examined the identity of these shared DEGs, we noted that more genes were downregulated with cytokine blockade, and most downregulated genes were involved in inflammatory and suppressive processes carried out by myeloid cells, such as *Cxcl2*, *Nlrp3*, *Arg1*, *Fn1,* and *Il1b* (Fig. 4B). CXCL2 was one of the most highly secreted chemokines by day 17 in our cytokine/chemokine panel (Fig. 1D). It has roles in attracting myeloid cells and other suppressive cells, including MDSCs [17]. While important for sensing pathogens, the dysregulated signaling of the NLRP3 inflammasome can induce excess inflammation and has a role in tumor pathogenesis [18]. Arginase 1 (*Arg1*) and fibronectin-1 (*Fn1*) are markers of immunosuppressive macrophages that potentiate tumor growth [5, 19]. Finally, the downregulation of *Il1b,* which was shared across blockade of each of the pro-inflammatory cytokines, suggested a shared set of genes regulated by these cytokines. Indeed, Gene Set Enrichment Analysis (GSEA) revealed significant depletion of both hallmark TNF and IL-6 signaling gene sets with any cytokine blockade (Fig. 4C). Blockade of one cytokine reduced the expression of the target cytokine, as well as the other two, with broader effects on cytokines in similar classes, such as *Osm* and *Lif* in the IL-6 family of cytokines (Fig. 4D). Taken together, these results suggested that blockade of any pro-inflammatory cytokine caused downregulation of shared inflammatory and suppressive pathways.

The list of shared, differentially expressed genes suggested that the major changes induced by cytokine blockade primarily affected myeloid cells, and more specifically macrophages. When cells were labeled by the number of shared DEGs that they expressed, those clusters most affected transcriptionally were indeed macrophages (Fig. 4E). The top shared, downregulated genes in these macrophages were similar to those shared by the total sample, including *Cxcl2, Nlrp3, Arg1, Fn1, and Il1b* (Fig. 4F). The shared transcriptional program also included reduced expression of genes involved in cell adhesion, chemotaxis, inflammation, and immunosuppression, including several transcription factors with known roles in regulating the inflammatory and immunosuppressive functions of macrophages [20–22]. This suggested that the main role that TNFα, IL-1β, IL-6 played in the tumor was exerted by and on macrophages. Overall, blocking these cytokines did not result in major changes to the cellular composition of the microenvironment, but rather caused interconnected changes to the transcriptional profile of macrophages. Taken together, these results suggested that pro-inflammatory cytokine blockade has a positive impact on anti-tumor immunity through the transcriptional modulation of intratumoral macrophages.

## Discussion

We previously established a way of generating stable B16 tumor masses by long-term treatment with *d*106S-IL12 virus [6]. To this end, we could identify potential immune mediators maintaining this balance between tumor elimination and escape. In this study, we profiled the tumor microenvironment for insight into factors that could result in escape from equilibrium, blockade of which might lead to full clearance of the tumor. Our discovery of high levels of the pro-inflammatory cytokines TNFα, IL-1β, and IL-6 led us to test if these cytokines aided in tumor destruction or promotion. Indeed, these pro-inflammatory cytokines antagonized the anti-tumor response initiated by IL-12 virotherapy, as their depletion resulted in complete cures of several mice, overcoming immune equilibrium.

Macrophages and MDSCs are the major producers of and responders to innate inflammatory cytokines. Although generally immunosuppressive in cancer, macrophage reprogramming can have a therapeutic benefit [23, 24]. Macrophages are adept at phagocytosis, and whether their phagocytosis of live tumor cells is enhanced upon blockade of TNFα, IL-1β, or IL-6 is unknown [25]. In our study we did not include an isotype control antibody, thereby leaving open the possibility that the transcriptional changes we observed in macrophages could result from potential engagement of Fc-receptors on macrophages from exogenously administered antibodies.

While often critical for the immune response to pathogens [26], pro-inflammatory cytokines (TNFα, IL-1β, and IL-6) play mixed roles in tumor pathogenesis [27]. Recombinant IL-6 has been shown to help mobilize a T-cell response against cancer [28]. Trans-signaling of IL-6 can enhance CD8^+^ T cell trafficking to the tumor, while also decreasing Treg presence [29] and IL-6 produced from dendritic cells (DCs) can increase T cell survival [30]. However, IL-6 blockade has also provided anti-tumor benefits in multiple preclinical models [10, 12, 31, 32]. IL-6 binding to tumor cells can directly support their growth and survival as well as enhance tumor aggressiveness by promoting vascular endothelial growth factor (VEGF) expression to support angiogenesis and metastasis [33]. IL-6 can also interfere with development of antigen-presenting cells, redirect myeloid progenitor cells to become suppressive cells, and block the maturation of DCs, preventing T-cell activation [33]. IL-6 and TNFα have both been implicated in cancer cachexia, and efforts to block these cachexins may also improve responses to cancer-directed therapies [34].

Although overwhelming evidence supports a role for IL-1β in promoting oncogenesis, the role of IL-1β in established cancers is less clear [8, 10]. Positive anti-tumor effects of recombinant IL-1β have been shown [35], and blockade of IL-1β by treatment with IL-1 receptor antagonist (IL-1Ra) impaired a cancer response by reducing Th1 and tumoricidal macrophage activation [36]. These beneficial effects are understandable as IL-1β has important roles in the activation, function, and trafficking of T cells [37]. However, IL-1β blockade has also provided benefits in treating cancer [38, 39]. We saw that with blockade of any cytokine, *Il1b* was one of the top shared downregulated genes. IL-1β can induce VEGF expression, which increases tumor aggressiveness [40]. The Canakinumab Anti-inflammatory Thrombosis Outcomes Study (CANTOS) trial examined whether patients treated with anti-IL1β (Canakinumab) for atherosclerosis had decreased incidence of cancer. Cancer incidence and mortality were significantly lower for those treated with anti-IL1β versus placebo, suggesting that anti-inflammatory cytokines may have a role in progression and severity of disease [8].

The definitive role of TNFα in the immune response to cancer is still debated. As its name suggests, TNFα was discovered for its ability to cause necrosis in tumors [41]. TNFα production by effector T cells has also been shown to lead to tumor cell death, particularly in tumors that are sensitized to extrinsic death pathways [42–44]. Inhibitors of cIAP1/2, also called SMAC mimetics, exploit this property by increasing production of TNFα from T cells, while additionally sensitizing tumors to cytokine-mediated cell death [44–48]. Additionally, TNFα has been used as a cancer therapeutic. Due to its systemic toxicity, methods of locally introducing TNFα to treat cancer have included isolated limb perfusion [49], viral vectors [50], and targeting peptides [51]. However, like IL-6, TNFα can also directly bind tumor cells to enhance survival and proliferation [7]. TNFα binding to CD8^+^ tumor-infiltrating T cells can cause their death and can increase the function and immunosuppression of MDSCs and Tregs [7]. Additionally, TNFα can cause a dedifferentiation of tumor cells, leading to loss of neoantigens and an increase in PD-L1 expression [7]. Indeed, TNFα blockade has been shown to pair effectively with PD-1 blockade in treating tumors in mice [52] and human clinical trials are underway (NCT05034536). Here we provide additional confirmation that TNFα blockade increases anti-tumor immunity and can pair with modalities beyond PD-1 blockade.

In cancer patients, immune-related adverse events have become more common, especially with combination immunotherapies, and can be treatment-limiting [53–55]. However, blockade of pro-inflammatory cytokines such as TNFα, holds promise as a method for reducing such adverse events, including checkpoint-induced colitis [55–57]. Clinically, TNFα blockade can be used to treat checkpoint-induced colitis concurrently with maintained dosing of the immunotherapy [13]. In checkpoint blockade colitis in humans, pathogenic IFNγ-producing CD8^+^ T cells are expanded from tissue-resident memory pools in the presence of expanded, albeit ineffective Tregs. Colitis-associated myeloid cells have higher expression of genes involved in innate inflammatory pathways, suggesting that pathology is driven by inflammasome activation and production of IL-1β, IL-6, and TNFα [57]. We therefore predict that innate inflammatory cytokine blockade may provide therapeutic benefit, by enhancing control of the tumor and preventing or alleviating colitis, one of the most common immune-related adverse events. Several clinical trials are underway or have completed testing the combination of pro-inflammatory cytokine blockade and checkpoint inhibitors (NCT03293784, NCT04121442, NCT03968419, NCT03821246, NCT04191421, NCT05034536). Correlates from these trials in humans will help advance our understanding of innate inflammatory cytokine blockade in the context of cancer therapeutics.

## Methods

### Cell and virus culture

B16F10 cells expressing human nectin-1 were generated previously [6]. The Vero-based E11 complementing cell line [58], which expresses ICP27 and ICP4, was used to grow virus. B16 and E11 cells were cultured in DMEM with 10% heat-inactivated FBS and 1% PenStrep. Viral stocks of both *d*106S and *d*106S-IL12 were grown and tittered on E11 complementing cells [58].

### Animals

C57BL/6J mice aged 6 to 8 weeks were purchased from Jackson Laboratories and housed in the Dana-Farber Cancer Institute Animal Resources Facility.

### Ethical approval

All animal experiments were performed in accordance with the DFCI IACUC-approved protocols (#14-019 and #14-037) and are in compliance with the NIH/NCI ethical guidelines for tumor-bearing animals.

### Tumor inoculations and *in vivo* experiments

B16^Nectin1^ cells were screened prior to *in vivo* use for murine pathogens, including mycobacteria (Charles River Laboratories). B16^Nectin1^ cells were cultured until 80–90% confluent, trypsinized, washed, and resuspended in Hank’s balanced salt solution (HBSS) at 2 × 10^6^ cells/mL. Mice were shaved and 250 µL (5 × 10^5^) cells were injected subcutaneously into the left flank. For survival experiments, tumor size was measured every 3–4 days by precision calipers. Mice were euthanized when tumor volume exceeded 2,000 mm^3^ or tumors developed ulcerations. Before injections, mice were randomized into treatment groups. Intratumoral injections of 30 µL PBS, *d*106S (1.5 × 10^7^ PFU/30 µL), or *d*106S-IL12 (1.5 × 10^7^ PFU/30 µL) were performed every 3 days. For cytokine blockade survival experiments, mice were injected i.p. with 200 µL (100 µg) of either anti-TNFα (BioXCell Clone XT3.11), anti-IL-1β (BioXCell Clone B122), or anti-IL6R (BioXCell Clone 15A7) starting on day 10 every 3 days for a total of six injections. For scRNA-seq, mice bearing tumors were injected intratumorally every 3 days with either PBS, *d*106S, or *d*106S-IL12 four times, starting at day 7 post-challenge. On day 10, mice received either no blockade or cytokine blockade targeting TNFα, IL-1β, or IL-6R for a total of three injections. On day 16 or 17, PBS or *d*106S/*d*106S-IL12 tumors, respectively, were collected for downstream scRNA-seq and flow cytometry.

### Single-cell RNA-sequencing and analysis

Tumors were collected and processed as previously described [6, 59]. Briefly, the tumor was excised, minced with scissors, and incubated in RPMI with tumor digestion enzymes (Miltenyi tumor dissociation kit, Cat. no. 130-096-730) for 30 min at 37°C. Tumors were filtered through a 40 µM strainer and cells were resuspended in FACS buffer (PBS with 2% FBS). Cells were counted with a hemocytometer and equal portions of cells from each mouse in a sample group were pooled before staining with anti-CD45-FITC (Biolegend clone 30-F11) and ZombieNIR (Biolegend #432105) and sorted for ZombieNIR- CD45^+^ events on into collection buffer (RPMI 1640, 25 mM HEPES, 10% FBS) on a BD FACS Aria II at the Dana-Farber Flow Sorting Core. Sorted cells were washed one time with 5 mL of PBS + 0.05% ultrapure BSA, counted via hemocytometer, and resuspended to a concentration of 1000 cells per microliter. Cell suspensions were loaded onto a 10X Chromium instrument with the Single Cell A Chip per manufacturer’s protocol with a targeted recovery of 5000 cells per sample. Library preparation was performed with the Chromium Single Cell 5’ Library and Gel Bead Kit (Part Number 1000006), and samples were sequenced on an Illumina HiSeq instrument with 2 × 150 bp sequencing.

The 10× CellRanger pipeline (v3.1.0) was used to align reads to the mm10 reference genome and generate a single-cell feature count matrix for each library using default parameters. The count matrices were imported for downstream analysis into R using the “Seurat” package (v3.1.5). Genes expressed in fewer than three cells were discarded from further analysis. Barcodes were classified as cells if they satisfied the following criteria: minimum of 500 reads detected in greater than 125 distinct genes, percentage of mitochondrial reads less than 2 SDs from the mean, and total reads less than 2 SDs of the mean. Counts from all samples were merged into one matrix, log-normalized, and scaled. The data were then subject to dimensionality reduction using principal component analysis (PCA) of the 2500 most variable genes. The top 43 principal components (cutoff determined by inflection on elbow plot) were selected for clustering and visualization. Clusters were identified first by constructing a shared nearest neighbor (SNN) graph based on each cell’s 75-nearest neighbors and then applying modularity refinement with the Louvain algorithm. Markers for each cluster were identified by comparing expressions using Model-based Analysis of Single-cell Transcriptomics (MAST) [60]. Two clusters consisted of doublet cells as determined by simultaneous expression of T-cell markers and B cell or macrophage markers with doubled RNA read counts per cell compared to pure lineage clusters; these clusters were excluded from analysis. Visualization of the data in two dimensions was done using t-distributed stochastic neighbor embedding (tSNE) at a perplexity of 100. In order to perform GSEA between two sets of samples with normalized gene expression matrices *A* and *B* and cell counts *n* and *m*, respectively, a rank was assigned for each gene *g* using the formula:


$$r_{g}=\log_{2}\;\left(\frac{\sum_{i=1}^{n}A_{g,i}}{n}+1\right)\;-\;\log_{2}\;\left(\frac{\sum_{i=1}^{m}B_{g,i}}{m}+1\right)$$

Enrichment scores were calculated from the ranked list using the R package ‘fgsea’ (v1.14.0). Gene sets were obtained from the Molecular Signatures Database (v7.2). Ribosomal genes were excluded from enrichment analysis.

### Flow cytometry

Tumors were collected and processed as above for scRNA-seq and stained with flow cytometry antibodies for 20 min at 4°C. Cells were washed once with PBS and fixed with 1% formalin in PBS before analysis, which was performed on a Sony Biotechnology SP6800 Spectral Analyzer and analyzed with the Sony Biotechnology SP6800 Software and FlowJo (Tree Star, Ashland, OR). Flow cytometry antibodies used in this study were purchased from BioLegend: anti-CD11b (clone M1/70), anti-CD11c (clone N418), anti-CD4 (clone GK1.5), anti-CD45 (clone 30-F11), anti-CD8 (clone 53-6.7), anti-Gr1 (clone RB6-8C5), and anti-Ly6C (clone HK1.4).

### Chemokine/cytokine analysis

Tumors treated with PBS, *d*106S, or *d*106S-IL12 were collected on day 10 and day 17 following one or four intratumoral injections, respectively, were snap frozen in liquid nitrogen. Frozen tumors were ground into a powder and tissue homogenate generated by addition of ice-cold RIPA lysis buffer (150 mM NaCl, 1% NP-40, 0.5% sodium deoxycholate, 0.1% SDS, 25 mM Tris) and 1:100 protease/phosphatase inhibitor cocktail (ThermoFisher). Homogenate was incubated by shaking for 30 min at 4°C and subsequently centrifuged at 14,000 × g for 15 min to yield clarified lysate. The clarified supernatant was subjected to a 32-plex cytokine/chemokine array (Eve Technologies). Expression of IL-13 was undetectable in all samples and is absent from the heatmap.

### Statistical analysis

All data were analyzed with GraphPad Prism or R. All data were presented as mean with SEM errors bars. Significance was determined with a one-way ANOVA and either Sidak’s, Tukey’s, or Dunnett’s multiple comparison tests; unpaired t-tests for pooled scRNA-seq flow cytometry; Wilcoxon signed rank test for comparing groups within scRNA-seq; Kaplan–Meier survival curves were analyzed using the log-rank test with a Bonferroni correction to adjust for multiple comparisons. Data were considered significant when *P* < 0.05; *P < 0.05, **P < 0.01, ***P < 0.001, ****P < 0.0001.

## Supplementary Material

ltad011_suppl_Supplementary_Figures

## Data Availability

Single-cell RNA-sequencing data are available at the Gene Expression Omnibus (GEO) under accession number: GSE160132. This paper does not report original code. Any additional information required to reanalyze the data reported in this manuscript is available from the corresponding authors upon request.

## Abbreviations:

DCs: Dendritic cells
HSV: Herpes simplex virus
IFN: Interferon
IL: Interleukin
MDSCs: Myeloid-derived suppressor cells
PBS: Phosphate-buffered saline
TNF: Tumor necrosis factor
