# Correction to: Blockade of innate inflammatory cytokines TNFα, IL-1β, or IL-6 overcomes virotherapy-induced cancer equilibrium to promote tumor regression

**DOI:** 10.1093/immadv/ltad019

**Published:** 2023-10-12

This is a correction to: Michael J Walsh, Lestat R Ali, Patrick Lenehan, Courtney T Kureshi, Rakeeb Kureshi, Michael Dougan, David M Knipe, Stephanie K Dougan, Blockade of innate inflammatory cytokines TNFα, IL-1β, or IL-6 overcomes virotherapy-induced cancer equilibrium to promote tumor regression, Immunotherapy Advances, Volume 3, Issue 1, 2023, ltad011, https://doi.org/10.1093/immadv/ltad011

The Conflict of interest statement of this manuscript has been corrected.

