# Supplementary material for: Blockade of innate inflammatory cytokines TNFα, IL-1β, or IL-6 overcomes virotherapy-induced cancer equilibrium to promote tumor regression
Source: Immunother Adv. 2023 Jul 3;3(1):ltad011. doi: 10.1093/immadv/ltad011 (PMC10349916; doi:10.1093/immadv/ltad011)
Supplement: ltad011_suppl_Supplementary_Figures [file ltad011_suppl_supplementary_figures.pdf]

Supplemetary Figure 1

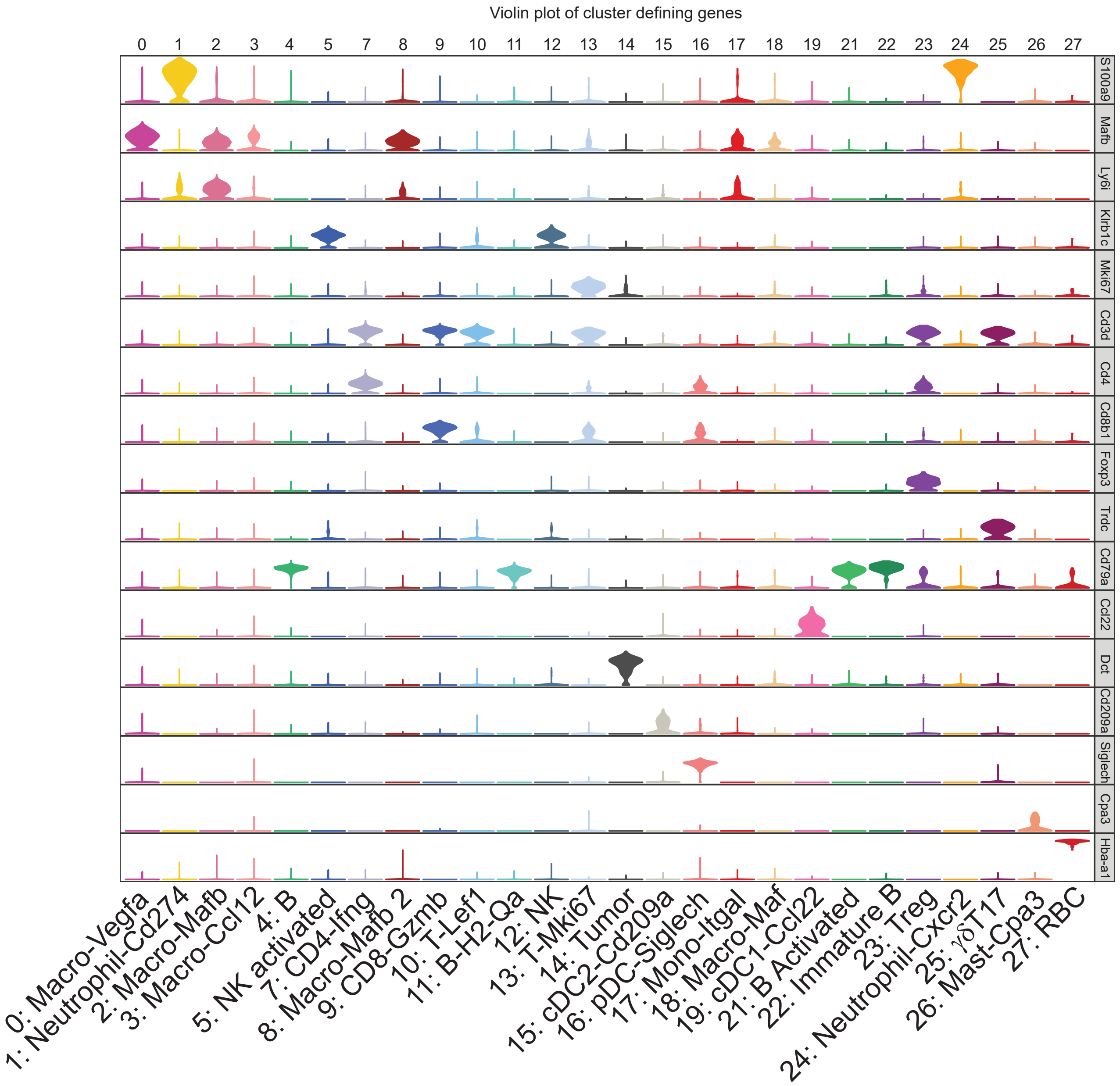

Supplementary Figure 1: Canonical gene expression from single-cell RNA-sequencing. Violin plot of canonical marker genes across scRNA-seq clusters from Figure 3A. Y-axis represents normalized gene expression.

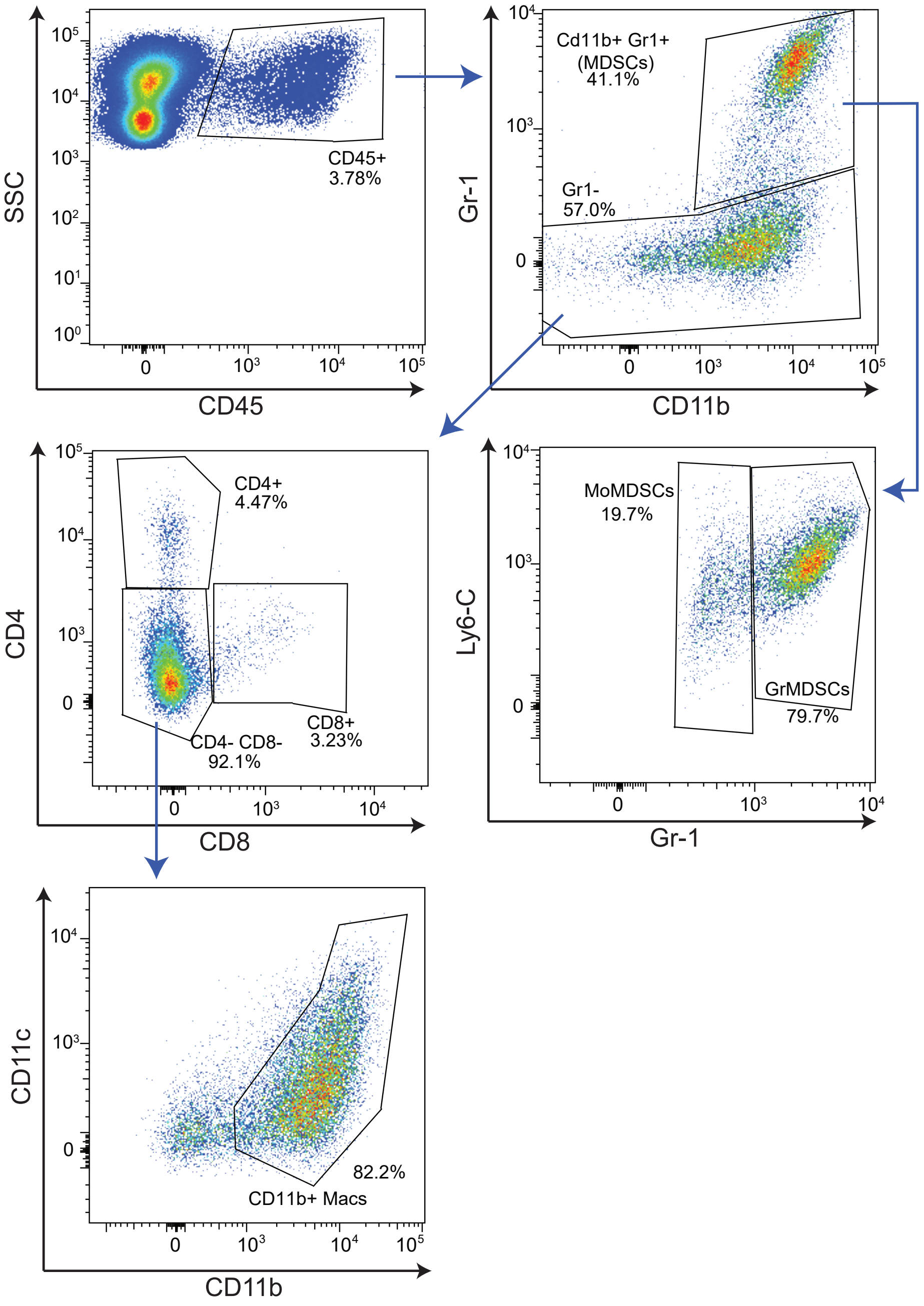

Supplementary Figure 2: Gating strategy for flow cytometry. Representative flow gating strategy for Figure 3D/E
